# On the Conservative Treatment of the Dental Pulp

**Published:** 1883-09

**Authors:** T. Charters White

**Affiliations:** England.


ARTICLE VII.

### ON THE CONSERVATIVE TREATMENT OF EXPOSED PULP.

BY T. CHARTERS WHITE, M. R. C. S., AND L. D. S., ENGLAND.

The most important development of dental science, of late years, has been the development of its conservative aspect as contra-distinguished from the medical plan, which may be defined as dragging teeth out "by the roots," and as every dental surgeon has had something to say upon it, and as I promised to contribute an article to our *Journal* on some subject of interest relating to our specialty, I hasten to redeem my promise by penning a few remarks on the Conservative Treatment of Exposed Pulp; not promising, however, anything new, but simply stating the plan I find sufficiently successful to recommend to others. Patients are constantly enjoined to come frequently under dental inspection, but enjoin and entreat, as you will, the majority keep away until more or less pain indicates the existence of something wrong, till advancing caries has, if not absolute exposed a dental pulp, at least laid it open to attacks of acid or to change of temperature; and then, having despised the injunction of their dental adviser, they expect him to make good the consequences of their neglect, or blame his want of skill if he fails to effect a perfect restoration. We explain that we can only do our best to remedy the evil, but cannot control all the whims of outraged nature; we can conscientiously employ all the aids which careful observation and experience direct, but our best endeavors may be thwarted by adverse conditions. An exposed pulp is a most "cantankerous" member to deal with, and requires as much humoring as a spoilt child or an exacting woman; you have to approach it with much tact and careful judgment, and have to bear in mind the various conditions under which it may be presented to your notice. It may come before you apparently well covered by softened dentine, but the dentine may be thin and permeable to irritating influences—or it may be thoroughly exposed and sphacelated; a variety of conditions may be found in the intervening range comprised within these limits, but whatever they may be they are generally amenable to the one treatment. A patient comes with a colorless or slightly yellow excavation in a tooth, complaining that it is only occasionally painful; you touch it and find the dentine soft and decalcified, but it may not have softened to any great extent; one or two decided cuts with the excavator may peel out the whole of the decalcified part, leaving a clean but highly sensitive surface. It is sufficient to coat this surface with a varnish composed of chloroform, mastic, and "Sanitas" oil and to plug it for a time with osteo-stopping to insure comfort to your patient, but if the advance of the disease has been greater, it may be necessary to interpose some non-conducting medium between the stopping and the bottom of the cavity. This may be readily found in those thin flanges of vulcanite left between the upper and lower surfaces of a flask after vulcanizing a denture. I always keep some of this by me for this purpose and find it an admirable resistant to thermal changes ; a piece of thin sheet-vulcanite is cut to a size suitable to the spot to be covered and steeped in the above-quoted varnish, and being soft is readily placed in the required position, when the stopping may be placed over it. In these cases " discretion is the better part of valor " and if a little dry decay be left at the bottom of the cavity, so that the nerves remain untouched, so much the better for your patient's comfort and the ultimate success of your treatment. The old notions of gold caps for this purpose were as unsatisfactory as they were unscientific ; for, being good conductors of heat and cold, they were unsuitable for protecting a sensitive surface. Quill caps were better, but not sufficiently manageable from being springy. But these caps of vulcanite can be shaped according to your requirements, and made either domed or used flat, as the necessities of the case may demand.

I have great faith in the application of " Sanitas " oil in the conservation of exposed pulps, having for the last four years given it a preference over carbolic acid. This oil, although named " Sanitas " as a trade designation, is a peroxide of hydrogen, and is a powerful antiseptic and deoderizer. I have seen mutton-chops which had been kept in a glass jar for over two years preserved from decomposition by a few drops of it placed with them ; also fresh herring, with all the pearly lustre they possessed in their ocean home preserved by the same means. I have seen barrels of serum from the blood of slaughtered animals (which is largely used in some processes of calico-dying) kept absolutely free from decomposition and odorless. It was seeing this which induced me to try it as an antiseptic in cases of exposed pulp, especially in those common cases of a partly decomposed pulp. In these cases it seems to arrest suppuration, while at the same time it is inocuous, and free from the charge of poisoning which has been laid at the door of carbolic acid. It is better to have half a nerve than none, and by using this we can keep half a pulp instead of wholly eradicating it, and there is a great chance of ultimately saving a tooth from alveolar michief. These are a few of the thoughts which arise from a consideration of this subject, and they are perhaps more likely to lead to practical results and discussion than an elaborate article. I com mend this method of treatment to the careful trial of that largely increasing section of our profession, the Conservative Dental Surgeon.—*The Journal of the British Dental Association.*

---

ARTICE VIII.

